# Simultaneous Detection and Quantification of Organic Acids and Furans in Lignocellulosic Biomass Hydrolysate Through High‐Performance Liquid Chromatography With Diode Array Detector

**DOI:** 10.1002/jssc.70216

**Published:** 2025-07-14

**Authors:** Patrizia Casella, Raffaele Loffredo, Maria Antonietta Rao, Federico Liuzzi, Isabella De Bari, Antonio Molino

**Affiliations:** ^1^ Italian National Agency for New Technologies, Energy and Sustainable Economic Development, Department of Sustainability, Division Sustainable Agri‐Food Systems, Laboratory Regenerative Circular Bioeconomy (ENEA‐SSPT‐AGROS‐BIOEC) Portici Italy; ^2^ Department of Agricultural Sciences University of Naples Fedrico II Portici Italy; ^3^ Italian National Agency for New Technologies, Energy and Sustainable Economic Development, Department of Energy Technologies and Renewable Sources, Division Bioenergy, Biorefinery and Green Chemistry, Laboratory Techniques and Processes for Biorefineries (ENEA‐TERIN‐BBC‐TPB) Rotondella (MT) Italy

**Keywords:** 5‐hydroxymethylfurfural, furfural, succinic acid, wheat straw

## Abstract

Lignocellulosic biomass is gaining attention as low‐cost renewable resources for sugars for fermentation and as a source of energy. Pretreatments and fermentation of these biomasses can generate organic acids and furans. Many liquid chromatography protocols have been developed for the analysis of these compounds. Organic acids are typically detected and quantified using diode array detector, while furans can be analyzed by using refractive index or ultraviolet detectors. In this work, the identification of succinic, lactic, formic, and acetic acids and two furans (5‐hydroxymethylfurfural and furfural) was performed by ultra‐high‐performance liquid chromatography coupled with diode array detector and ion chromatography columns. Different chromatographic conditions were tested by varying the column temperature and the flow rate of sulfuric acid 5 mM. Calibration curves, peak resolution, limit of detection, and limit of quantification were calculated using analytical standards at known concentrations for each compound. The accuracy was evaluated by the recovery of the compounds in wheat straw hydrolysate. For succinic acid, the best condition was at a flow rate of 0.6 mL/min and a column temperature of 60°C while formic and lactic acids and furans were better recovered at 1.0 mL/min and 60°C.

## 1 Introduction

The use of fossil fuels in chemical production processes is no longer sustainable, as their use entails several issues, including increasing cost and significant environmental damage created by the greenhouse gas emission. In recent years, to reduce the use of fossil fuels, a shift to renewable feedstock has been promoted, thus supporting the transition to a circular economy [1]. Lignocellulosic biomass can be a suitable low‐cost feedstock for bio‐based processes, being freely available in high quantities with an annual global production estimated at around 220 billion tons from various production chains [2], and possessing a high concentration of sugars for fermentation. The average composition of lignocellulosic biomass is mainly composed of cellulose 23%–50% (dry basis [d.b.]), hemicellulose 12%–29% (d.b.), and lignin 13%–31% (d.b.). The lignocellulosic matrix needs pretreatments to break down and to hydrolyze cellulose and hemicellulose into fermentable sugars for the microorganisms employed in fermentation processes [3]. But during the severe conditions of pretreatments, that is, high temperature 160°C–220°C or during fermentations, several compounds can be generated, such as furan derivatives and weak acids. Succinic acid (SA), lactic acid (LA), and formic acid (FA) are intermediate and final products of several biochemical reactions during fermentation. Among the furans, 5‐hydroxymethylfurfural (HMF) and furfural (FU) originated from the degradation of the monomers, such as glucose and xylose, while the hydrolysis of acetyl group in hemicellulose caused the formation of acetic acid (AA) (Figure S1) [4].

To date, several HPLC methods have been used to detect and quantify organic acids and furans (Table S1). To analyze HMF and FU, the HPLC‐UV method was used at a wavelength of 290 nm, an RP‐18 column (at 20°C), and an isocratic method, with a water–acetonitrile mixture (9:1 v/v) as mobile phase and 1.4 mL/min flow rate [5]. Instead, a different analytical method to analyze the same compounds employed an ion‐exclusion column coupled with a refractive index detector (RID), at temperatures of 60°C and 45°C, respectively, for HMF and FU and 5 mM H_2_SO_4_ at the flow rate of 0.6 mL/min [6]. Furfuryl alcohol and HMF and their carboxylic acid derivatives were quantified in coffee grounds by a liquid chromatography method using C18 column at 25°C by using gradient mobile phase of AA in water and methanol [7]. In a recent work, for organic acids, an isocratic method (H_2_SO_4_ 5 mM as eluent) was employed by using an ion‐exclusion column and RID, while for the analysis of furans, a C18 column and a gradient elution method (0.2% v/v FA and 100% v/v acetonitrile) were used [8]. For the detection and quantification of SA, FA, and AA, an H^+^ ion‐exchange column (at 50°C) and a diode array detector (DAD) were used at a wavelength of 210 nm, 0.6 mL/min as flow rate, and isocratic mobile phase H_2_SO_4_ 10 mM [9]. To detect organic acids, another method employed a reverse‐phase silica column at 30°C coupled with DAD, at 210 nm [10]. A mix of Na_2_SO_4_ 0.2 M with 0.55 mL/L of methanesulfonic acid was used as mobile phase, at a flow rate of 0.3 mL/min. An optimization test for a simultaneous detection method of FA, levulinic acid, HMF, and FU was performed using DAD and an H^+^ ion‐exchange column while testing different mobile phases (0.1% TFA v/v, H_2_SO_4_ 5 mM, and acetonitrile), column temperatures, and flow rates []. Furthermore, a chromatographic method for the simultaneous quantification of fatty acids and nonvolatile organic acids, such as FA, LA, and SA, in *Hevea brasiliensis* latex was developed by using KH_2_PO_4_ and acetonitrile as mobile phases, HPLC‐UV and a C18 column [12]. A simultaneous detection protocol for organic acids and furan derivatives would be very useful, as it would reduce both the time required for analysis and the amount of reagents used as eluents, as well as decrease the energy consumption of the instrumentation.

In this study, the chromatographic of SA, LA, FA, and AA and HMF and FU that are principally contained in lignocellulosic biomass hydrolysate were optimized by µHPLC‐DAD. An H^+^ ion‐exchange column, DAD, and H_2_SO_4_ as eluent were used for this purpose. Several tests were performed, testing different combinations of column temperature (40°C to 60°C) and flow rate (0.6 and 1 mL/min). The linearity of the calibration, peak resolution, limit of detection (LOD), and limit of quantification (LOQ) were evaluated, and recovery of these compounds was evaluated from the hydrolysate.

## 2 Material and Methods

### 2.1 Chemicals and Materials

The analytical standards used for SA, LA, FA, AA, HMF, and FU were purchased from Sigma‐Aldrich (Darmstadt, Germany) (> 99% purity). LC‐MS grade water and H_2_SO_4_ were employed for the preparation of the mobile phase. Wheat straw hydrolysate used as a study sample was provided and treated in ENEA Research Center of Trisaia (Rotondella, MT, Italy). The characterization of the hydrolysate was also provided (Table S2).

### 2.2 Sample Preparation

Standard stock solution was prepared mixing all the analytical standards at known concentration and then was diluted in LC‐MS grade water. Calibration curves ranged from 20 to 200 mg/L of organic acids, from 0.8 to 8 mg/L of HMF, and from 4 to 40 mg/L of FU. For the accuracy tests, wheat straw hydrolysate was filtered at 0.22 µm (PTFE syringe filter, Whatman plc., Maidston, UK) and then it was used at 1:20 dilution.

### 2.3 µHPLC‐DAD Optimized Methods

An µHPLC‐DAD (1290 Infinity II, Agilent Technologies Inc., Santa Clara, USA) and an H^+^ Ion Exclusion/Ligand Exchange column (Hi‐Plex H, 7.7 × 300 mm, 8 µm, Agilent Technologies Inc., Santa Clara, USA) were used. For protocol optimization, samples were analyzed at three different column temperatures (40°C, 50°C, and 60°C), in isocratic condition at two flow rates 0.6 and 1 mL/min. Sulfuric acid (5 mM) was used for mobile phase and 20 µL ijection volume was set. DAD has been set at 210 nm to detect organic acids, at 276 nm to detect FU, and at 284 nm to detect HMF. To evaluate the performance of µHPLC‐DAD method, different concentrations of analytical standards were prepared as reported in Section 2.2 to test the linearity, the resolution of the peaks, LOD, and LOQ.

The regression coefficient (*R* ^2^) measured the correlation between the concentration of the analytes and the peak areas and it was accepted for value > 0.99.

Resolution (*R_S_*) was calculated through Equation (1):

(1) $$R_{S}=\frac{2\left(t_{R2}-t_{R1}\right)}{W_{1}+W_{2}}$$

where $t_{R2}$ is the retention time of peak 2, $t_{R1}$ is the retention time of peak 1, *W*_1_ is the width of peak 1, and *W*_2_ is the width of peak 2.

LOD and LOQ were calculated from the standard deviations of the *y*‐intercept (from the equation of the calibration curve) and the slope according to Equations (2) and (3), respectively:

(2) $$\mathrm{LOD}=3.3\frac{\mathrm{SD}}{S}$$

(3) $$\mathrm{LOQ}=10\frac{\mathrm{SD}}{S}$$

where SD is the standard deviation of the *y*‐intercept (from the equation of the calibration curve) and *S* is the slope of the calibration curve.

For the recovery (*R*%), a standard solution was prepared adding three concentrations of the analytes (50–66.6–100 mg/L of SA, LA, FA, and AA; 1–2.1–3 mg/L of HMF; and 19–20.6–24 mg/L of FU) to a diluted hydrolyzed wheat straw. The recovery was calculated by Equation (4):

(4) $$R\left(\%\right)=\frac{A-B}{C\;}\;\times 100\%$$

where *A* is the measured concentration of the sample with the standard added, *B* is the original concentration of the analyte in the sample (wheat straw hydrolysate), and *C* is the theoretical concentration of the added standard.

All tests were conducted in triplicate and the standard deviation was calculated.

## 3 Results and Discussion

For all tested chromatographic conditions, calibration curves were plotted by using known concentrations of compounds and peak areas (Figures S2–S7). Overall, the linearity of calibration curves of the compounds well fitted to the experimental data and the *𝑅* ^2^ value was > 0.99 under almost all conditions (Table S3).

Flow rate and temperature appeared to slightly affect the linearity of SA and LA. At 0.6 mL/min flow rate and 40°C, *R* ^2^ value was 0.98 for SA while for LA the lower *R* ^2^ was recorded at 1.0 mL/min flow rate and 40°C. The linearity of the HMF and FU curves were very good under most conditions (*R* ^2^ > 0.99) except at 1 mL/min and 50°C (*R* ^2^ < 0.99). A good peak resolution was observed for all compounds at all chromatographic conditions (*R_S_* > 1.5) as shown in the chromatographs of the two conditions 0.6 mL/min 60°C and 1.0 mL/min 60°C (Figures S8 and S9). In Table 1, the LOD and LOQ values have been reported for all the organic acids.

**TABLE 1:** LOD and LOQ expressed as mg/L of organic acids.

**Chromatographic conditions**	**SA** **(mg/L)**		**LA** **(mg/L)**		**FA** **(mg/L)**		**AA** **(mg/L)**	
	**LOD**	**LOQ**	**LOD**	**LOQ**	**LOD**	**LOQ**	**LOD**	**LOQ**
0.6 mL/min—40°C	12.50	38.00	1.80	5.60	0.39	1.19	2.06	6.24
0.6 mL/min—50°C	2.00	5.90	1.40	4.10	0.90	2.80	1.50	4.50
0.6 mL/min—60°C	0.56	1.70	1.69	5.12	1.00	3.10	0.95	2.89
1 mL/min—40°C	4.50	13.70	2.90	8.90	0.69	2.08	1.61	4.88
1 mL/min—50°C	1.90	5.90	2.20	6.60	0.43	1.31	1.87	5.67
1 mL/min—60°C	10.90	33.20	7.80	23.70	1.40	4.25	4.75	14.39

Keeping the flow rate at 0.6 mL/min, the LOD of SA dropped from 12.50 to 0.56 mg/L at the increase of the column temperature from 40°C to 60°C. At 1.0 mL/min and 50°C, the lowest LOQ value of SA was detected (5.90 mg/L). At 0.6 mL/min and 60°C, the lowest LOD and LOQ values of AA were observed. The best values of LOD (1.40 mg/L) and LOQ (4.10 mg/L) of LA were recorded at 0.6 L/min and 50°C, while at 0.6 L/min and 40°C were the best conditions of LOD and LOQ of FA. The LOD and LOQ values of SA, LA, FA, and AA obtained in this work were lower than the values reported by authors who used an HPLC‐UV, a C18 silica column at 30°C, and KH_2_PO_4_ and acetonitrile as the mobile phase [12]. Specifically, the LOD and LOQ values of SA achieved at 60°C and 0.6 mL/min were almost 10 times lower than the values reported by Arti et al. (2022) (30.68 mg/L [LOD] and 103.51 mg/L [LOQ], respectively) [12]. A lower LOD and LOQ of FA were obtained in this work respect to the HPLC‐DAD method by using 0.1% (v/v) TFA as mobile phase, column temperature of 60°C and stepwise flow rate at 0.6 mL/min (0–25 min) for acids and at 1 mL/min (26–50 min) for HMF and FU [11].

LOD and LOQ values of HFM and FU were lower in all the tested conditions, indicating that they were easily detectable and quantified even at low concentrations, as possible to see in Table 2.

**TABLE 2:** LOD and LOQ expressed as mg/L of furans.

**Chromatographic conditions**	**HMF** **(mg/L)**		**FU** **(mg/L)**	
	**LOD**	**LOQ**	**LOD**	**LOQ**
0.6 mL/min—40°C	0.02	0.06	0.05	0.14
0.6 mL/min—50°C	0.05	0.14	0.02	0.07
0.6 mL/min—60°C	0.02	0.07	0.05	0.14
1 mL/min—40°C	0.02	0.06	0.04	0.12
1 mL/min—50°C	4 × 10^−3^	0.01	0.09	0.27
1 mL/min—60°C	0.01	0.03	0.02	0.06

At the temperature of 50°C and 1.0 mL/min, the LOD and LOQ of HMF decreased to 4 × 10^−3^ and 0.01 mg/L, respectively. At 1.0 mL/min and 60°C, the best values of LOD and LOQ of FU were recorded (0.02 and 0.06 mg/L). In this work, LOD and LOQ of the two furans were lower than the values of LOD (HMF 1 mg/L and FU 3.46 mg/L) and LOQ (HMF 2.91 mg/L and FU 1.25 mg/L) obtained by Saengsen et al. (2022) [11]. Furthermore, the LOD and LOQ values obtained in this work were also lower than those observed by Li et al. (2017) [6] that tested an HPLC method using a C18 at 35°C, 20% methanol (v/v) 0.8 mL/min and that compared this method with the method of NREL. The LOD and LOQ obtained for HMF were also higher than that reached by using another HPLC‐DAD method that was developed for the simulated quantification of four furans contained in coffee grounds using a C8 column at 25°C, an elution gradient with 0.1% AA and methanol at 25°C at 0.5 mL/min [7].

**FIGURE 1:**
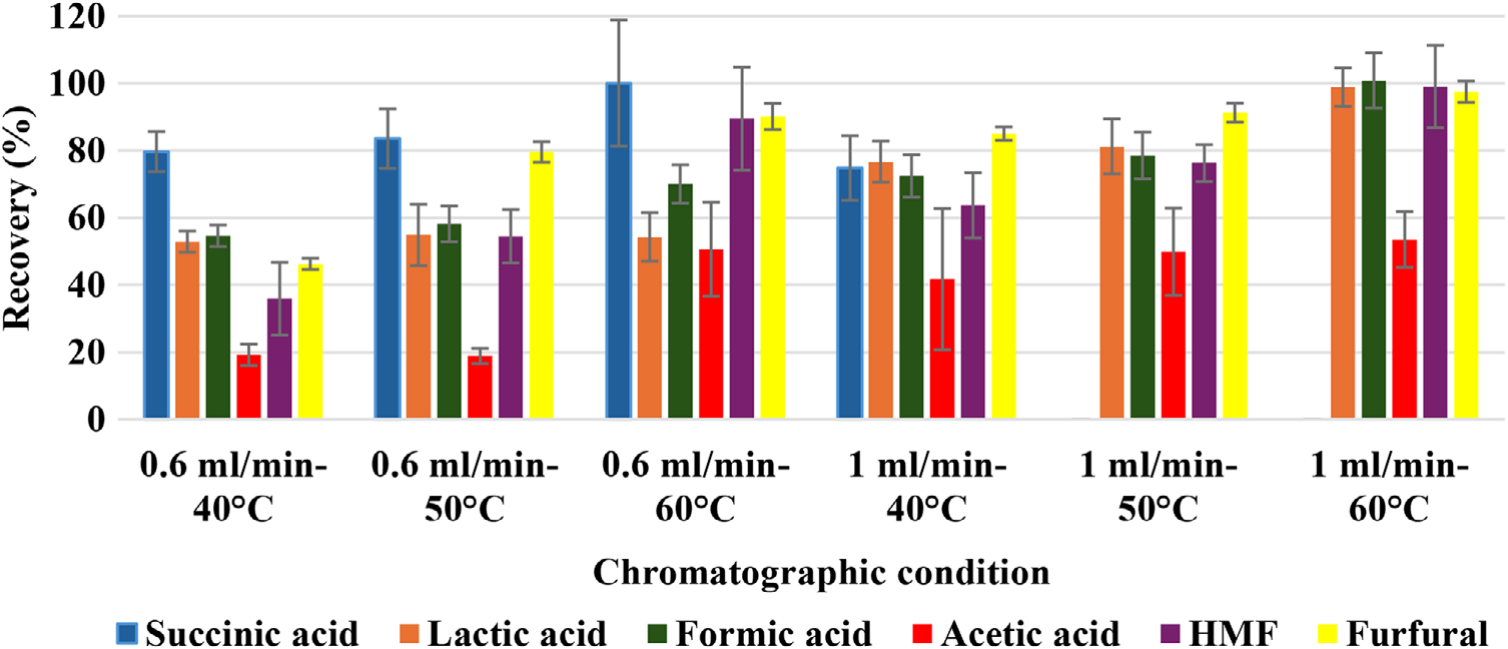
Recovery percentage (%) obtained for each compound using a hydrolyzed wheat straw.

In Figure 1, the recovery of all the analyzed compounds was reported to evaluate the accuracy of an analytical method. This parameter measured the efficiency of quantification of the compounds that were added in the study sample, the hydrolyzed wheat straw that interfered with the accuracy of the method as shown in Figures S10 and S11. After the addition of known quantities of acids (SA, LA, FA, and AA), furfural, and HMF to the wheat straw hydrolysate, the same sample was analyzed under all chromatographic conditions in order to assess the accuracy of the quantification of the added quantities (expressed as recovery percentage [%]).

The recovery of SA was lower at 0.6 mL/min and 40°C (79.66% ± 5.92). The best separation and quantification of SA in wheat straw was observed at 0.6 mL/min and 60°C (Figure S10) with a recovery of 100% ± 18.77. Instead, at flow rate of 1 mL/min at 40°C, the recovery of SA decreased significantly, going to 74.77% ± 9.65, and it was extremely overestimated (> 150%) at 50°C and 60°C. At the column temperature of 40°C and flow rate at 0.6 mL/min, the recovery was only 19.16% ± 3.20, indicating that AA was not easily separable under these conditions. Increasing the temperature to 50°C, the recovery did not seem to have improved, but at 60°C, the recovery reached the value of 50.6%. Even by varying the flow rate, the recovery did not significantly improve respect to 50.6% ± 14.03 (at 0.6 mL/min and 60°C), with a maximum value of AA recovery observed at 1.0 mL/min and 60°C (53.55% ± 8.32). This lack of difference on the recovery of AA was also observed in terms of quality by comparing chromatograms obtained at 60°C and 0.6 mL/min (Figure S10) to 1.0 mL/min (Figure S11). The recovery of LA and FA was similar, and the best condition was observed at 1.0 mL/min and 60°C (98.8% LA and 100% FA). In this condition, the recovery of HMF and FU was also improved, with a result near the 100% (99 ± 12.26 and 97.5% ± 3.16, respectively), comparable to that obtained (99% for both) by using an HPLC‐RID method, and a C18 column to detect HMF and furfural in biomass hydrolysate [6]. In this work, the recovery of FA was better at 1.0 mL/min at 60°C with respect to the value (94.22%) obtained by using µHPLC‐DAD by 0.1% TFA at 60°C and 0.6 mL/min [11].

Although the µHPLC‐DAD method tested in this work was not very different from the current NREL method using the RID detector^13^, the authors demonstrated that despite the interference of the wheat straw hydrolysate, the best accuracy for the analysis of LA, FA, HMF, and FU was at the flow rate of 1.0 mL/min and 60°C which also allowed to reduce the analytical times from 50 to 35 min (Figures S9–S11). Therefore, the best condition to analyze SA in hydrolysate wheat straw remained 0.6 mL/min and 60°C (Figures S8–S10).

## 4 Conclusion

An improvement of the methodology of the simultaneous chromatographic analysis of two distinct groups of compounds, organic acids and furans, has been successfully achieved. This advancement marked a significant step forward in optimizing the analytical process for these compounds, demonstrating the potential for more efficient and reliable analysis on lignocellulosic biomass hydrolysate. However, despite this progress, further optimization is possible to refine the methodology and enhance its performance. One notable challenge remained the poor recovery of acetic acid, which was a weak point in this methodology. Addressing this issue will be crucial to ensuring robustness and accuracy, and further research and adjustments in the experimental conditions may be necessary to overcome this limitation.

## Supporting information




**Supporting information File 1**: jssc70216‐sup‐0001‐SuppMat.docx
