# Supplementary material for: Simultaneous Detection and Quantification of Organic Acids and Furans in Lignocellulosic Biomass Hydrolysate Through High‐Performance Liquid Chromatography With Diode Array Detector
Source: J Sep Sci. 2025 Jul 14;48(7):e70216. doi: 10.1002/jssc.70216 (PMC12260114; doi:10.1002/jssc.70216)
Supplement: Supplementary file 1 — Supporting information File 1: jssc70216‐sup‐0001‐SuppMat.docx [file JSSC-48-e70216-s001.docx]

**Simultaneous detection and quantification of organic acids and furans in lignocellulosic biomass hydrolysate through high performance liquid chromatography with diode array detector**

Patrizia Casella^1,*^, Raffaele Loffredo^1,2^, Maria Antonietta Rao^2^, Federico Liuzzi^3^, Isabella De Bari^3^, Antonio Molino ^1^,^*^

^1^ Italian National Agency for New Technologies, Energy and Sustainable Economic Development - Division Sustainable, Agri-Food Systems, Laboratory Regenerative Circular Bioeconomy (ENEA-SSPT-AGROS-BIOEC, Piazzale Enrico Fermi 1, 80055 Portici (NA), Italy

^2^ Department of Agricultural Sciences, University of Naples, Federico II, Via Università 100, 80055 Portici (NA), Italy

^3^ ENEA, Italian National Agency for New Technologies, Energy and Sustainable Economic Development - Department of Energy Technologies and Renewable Sources, Processes and Technologies for Biorefineries and Green Chemistry Division, Techniques and Processes for Biorefineries laboratory (ENEA-TERIN-BBC-TBP), Strada Statale Jonica km 419 + 500, 75026 Rotondella (MT), Italy

* Email: [antonio.molino@enea.it](mailto:antonio.molino@enea.it); patrizia.casella@enea.it

**Keywords:** succinic acid, wheat straw, furfural, 5-hydroxymethylfurfuraldehyde,

1. **Supplementary material**


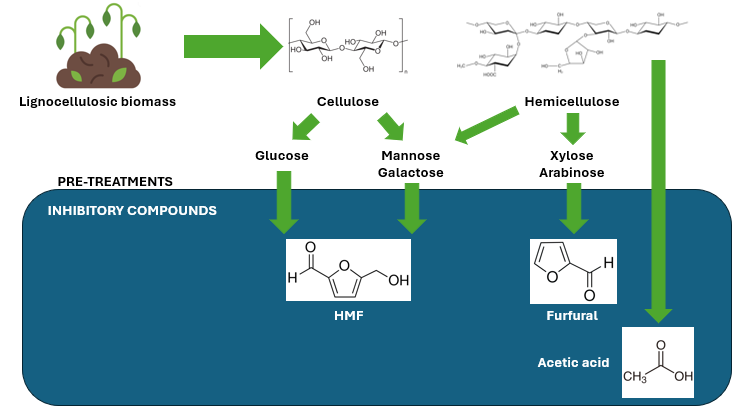


**Figure S1. Lignocellulosic biomass structure and inhibitory by-products formed after pretreatment (Modified from Tramontina et al. 2020) [4]**

**Table S1.** Characterization of the hydrolyzed wheat straw (glu = glucose, xyl = xylose, xilog = xyloglucan; AA; FU; HMF)

| **Concentration (g/l)** | | | | | |
| --- | --- | --- | --- | --- | --- |
| **glu** | **xyl** | **xylog** | **AA** | **FU** | **HMF** |
| 22.1 | 12 | 0.07 | 1.68 | 0.48 | 0.07 |

**Table S2.** Comparison of operative chromatographic conditions

| **Starting matrix** | **Instrument** | **Column** | **T° column** | **Detector** | **Mobile phase** | **Injection volume** | **Flow rate** | **Detected compounds**  **(wavelenght used)** | **Reference** |
| --- | --- | --- | --- | --- | --- | --- | --- | --- | --- |
| - Wheat straw | uHPLC | H^+^ ion-exchange  (7.7 x 300 mm,  8 µm) | 40 °C  50 °C  60 °C | DAD | - 0.005 M H_2_SO_4_ | 20 μL | 0.6 ml/min  1 ml/min | - Succinic acid (210 nm) - Lactic acid (210 nm) - Formic acid (210 nm) - Acetic acid (210 nm) - HMF (284 nm) - Furfural (276 nm) | This work |
| - Beech wood - Spruce wood - *Miscanthus x giganteus* | HPLC | - RP-18 column (for furan) - Aminex HPX-87H (for acids) | 20 °C  (for furan)  25 °C  (for acids) | UV (for furans)  DAD (for acids | - Water / acetonitrile (9:1 v/v) for furans - 0.004 mol/l of H_2_SO_4_ for acids | ND | 1.4 ml/min  (for furan)  0.65 ml/min  (for acids) | - Formic acid - Acetic acid (ND) - Levulinic acid (ND) - HMF (290 nm) - Furfural (290 nm) | [5] |
| - Corn stover | HPLC | C18  (4.6 × 150 mm,  5 μm) | 35 °C | RID | - 20% methanol (v/v) | 20 μL | 0.8 ml/min | - HMF (277 nm) - Furfural (268 nm) | [6] |
| - Waste liquid produced from alkali pretreatment of sugarcane bagasse (SCB) | HPLC | Shodex sugar SH1011 column  (8.00 mm ID × 300 mm) | 50 °C | RID | - 0.005 M H_2_SO_4_ |  | 0.5 ml/min | - Succinic acid - Formic acid - Acetic acid | [8] |
| - Wheat straw | uHPLC | H^+^ ion-exchange  (7.7 x 300 mm,  8 µm) | 50 °C | DAD | - 0.01 M H_2_SO_4_ | 20 μL | 0.6 ml/min | - Succinic acid (210 nm) - Lactic acid (210 nm) - Formic acid (210 nm) - Acetic acid (210 nm) | [9] |
| - Pomegranate molasses - Balsamic vinegar - Apple cider vinegar | HPLC | Reverse phase silica (4.0 × 250 mm, 5 µm) | 30 °C | DAD | - 0.2 M Na_2_SO_4_ with 0.55 ml of methanosulfonic acid added per liter | 2 μL | 0.3 ml/min | - Formic acid (210 nm) - Acetic acid (210 nm) - Citric acid (210 nm) - Levulinic acid (210 nm - Valeric acid (210 nm) - Diformylfuran (210 nm) - HMF (210 nm) - Furfural (210 nm) | [10] |
| - Oil palm empty fruit bunch | HPLC | H^+^ ion-exchange  (7.7 x 300 mm,  8 µm) | 40 °C  50 °C  60 °C | DAD | - 0.1% TFA v/v - H_2_SO_4_ 0.005 M - acetonitrile | 20 μL | 0.6 ml/min  1 ml/min | - Formic acid (210 nm) - Levulinic acid (210 nm) - HMF (266-276-284 nm) - Furfural (266-276-284 nm) | [11] |
| - Latex serum - Latex distillates | HPLC | C18-silica ( 150 × 4.6 mm id, 5 μm particle size;  ALLTECH, USA) | 30 °C | UV | - KH_2_PO_4_ - Acetonitrile | 20 μL | 1 ml/min | - Formic acid - Acetic acid - Propionic acid - Butyric acid - Valeric acid - Oxalic acid - Malic acid - Lactic acid - Citric acid - Succinic acid | [12] |

**Figure S2.** Calibration curves and equations succinic acid

| **Succinic acid** | | |
| --- | --- | --- |
| **Chromatographic condition** | **Equation** | **R^2^** |
| 0,6 ml/min - 40°C | y = 0,7933x-7,7777 | 0.983 |
| 0,6 ml/min - 50°C | y = 1,4281x-13,247 | 0.9959 |
| 0,6 ml/min - 60°C | y = 2,855x-15,94 | 0.9941 |
| 1 ml/min - 40°C | y = 0,8639x-14,897 | 0.9918 |
| 1 ml/min - 50°C | y = 0,849x-2,2764 | 0.999 |
| 1 ml/min - 60°C | y = 1,3076x-8,727 | 0.9967 |

**Figure S3.** Calibration curves and equations lactic acid

| **Lactic acid** | | |
| --- | --- | --- |
| **Chromatographic condition** | **Equation** | **R^2^** |
| 0,6 ml/min - 40°C | y = 4,9314x-44,641 | 0.9945 |
| 0,6 ml/min - 50°C | y = 4,5396x-8,5403 | 0.9976 |
| 0,6 ml/min - 60°C | y = 5,948x-21,974 | 0.9981 |
| 1 ml/min - 40°C | y=3,2346x-25,005 | 0.9935 |
| 1 ml/min - 50°C | y = 2,9364x-18,491 | 0.9952 |
| 1 ml/min - 60°C | y = 2,7117x-11,018 | 0.9954 |

**Figure S4.** Calibration curves and equations formic acid

| **Formic acid** | | |
| --- | --- | --- |
| **Chromatographic condition** | **Equation** | **R^2^** |
| 0,6 ml/min - 40°C | y = 10,415x-15,504 | 0.9988 |
| 0,6 ml/min - 50°C | y = 10,559x-32,873 | 0.9980 |
| 0,6 ml/min - 60°C | y = 12,407x - 21,267 | 0.9984 |
| 1 ml/min - 40°C | y = 5,1748x-0,922 | 0.9985 |
| 1 ml/min - 50°C | y = 5,9989x-24,077 | 0.9974 |
| 1 ml/min - 60°C | y = 6,3348x-31,293 | 0.9972 |

**Figure S5.** Calibration curves and equations acetic acid

| **Acetic acid** | | |
| --- | --- | --- |
| **Chromatographic condition** | **Equation** | **R^2^** |
| 0,6 ml/min - 40°C | y = 15,599x+33,822 | 0.9969 |
| 0,6 ml/min - 50°C | y = 12,454x-14,768 | 0.9982 |
| 0,6 ml/min - 60°C | y = 11,082x - 20,425 | 0.9981 |
| 1 ml/min - 40°C | y = 9,6459+12,609 | 0.9974 |
| 1 ml/min - 50°C | y = 7,204-13,89 | 0.9984 |
| 1 ml/min - 60°C | y = 7,6818X+7,1985 | 0.9985 |

**Figure S6.** Calibration curves and equations HMF

| **HMF** | | |
| --- | --- | --- |
| **Chromatographic condition** | **Equation** | **R^2^** |
| 0,6 ml/min - 40°C | y = 1506,5x-72,688 | 0.9996 |
| 0,6 ml/min - 50°C | y = 1421,2x-140,14 | 0.9985 |
| 0,6 ml/min - 60°C | y = 1503,4x-133,76 | 0.9986 |
| 1 ml/min - 40°C | y = 979,68x-52,872 | 0.999 |
| 1 ml/min - 50°C | y = 982,73x-115,9 | 0.984 |
| 1 ml/min - 60°C | y = 956,1x-105,31 | 0.9985 |

**Figure S7.** Calibration curves and equations furfural

| **Furfural** | | |
| --- | --- | --- |
| **Chromatographic condition** | **Equation** | **R^2^** |
| 0,6 ml/min - 40°C | y = 1443,1x-728,1 | 0.9991 |
| 0,6 ml/min - 50°C | y = 1574,3x-923,01 | 0.9977 |
| 0,6 ml/min - 60°C | y = 1614,8x-808,43 | 0.9983 |
| 1 ml/min - 40°C | y = 979,82x-323,98 | 0.999 |
| 1 ml/min - 50°C | y = 976,69x-493,28 | 0.981 |
| 1 ml/min - 60°C | y = 992,61X-462,55 | 0.9982 |

**Table S3. R^2^ value for the tested chromatographic conditions**

| **Chromatographic conditions** | **SA**  **(20-200 mg/l)** | **LA**  **(20-200 mg/l)** | **FA**  **(20-200 mg/l)** | **AA**  **(20-200 mg/l)** | **HMF**  **(0.8-8 mg/l)** | **FU**  **(4-40 mg/l)** |
| --- | --- | --- | --- | --- | --- | --- |
| 0.6 ml/min – 40 °C | 0.983 | 0.995 | 0.999 | 0.997 | 0.999 | 0.999 |
| 0.6 ml/min – 50 °C | 0.996 | 0.998 | 0.998 | 0.998 | 0.999 | 0.998 |
| 0.6 ml/min – 60 °C | 0.994 | 0.998 | 0.998 | 0.998 | 0.999 | 0.998 |
| 1 ml/min – 40 °C | 0.992 | 0.994 | 0.999 | 0.997 | 0.999 | 0.999 |
| 1 ml/min – 50 °C | 0.999 | 0.995 | 0.997 | 0.998 | 0.984 | 0.981 |
| 1 ml/min – 60 °C | 0.997 | 0.995 | 0.997 | 0.999 | 0.999 | 0.998 |

**Figure S8.** Chromatograms of the chromatographic conditions 0.6 ml/min at 60°C of standard solution

**Figure S9.** Qualitative chromatograms of the chromatographic conditions 1.0 ml/min at 60°C of standard solution


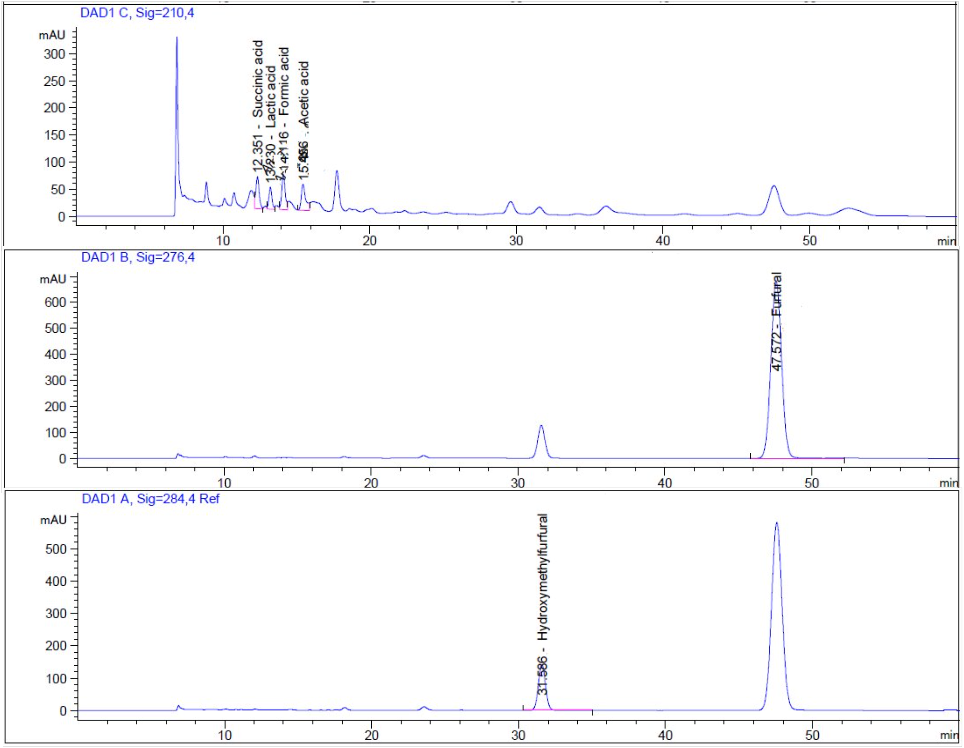
**Figure S10.** Chromatograms of the chromatographic conditions 0.6 ml/min at 60°C of the study sample wheat straw hydrolysate supplemented with 100 mg/l of succinic, lactic, formic and acetic acid, 3 mg/l HMF and 24 mg/l furfural


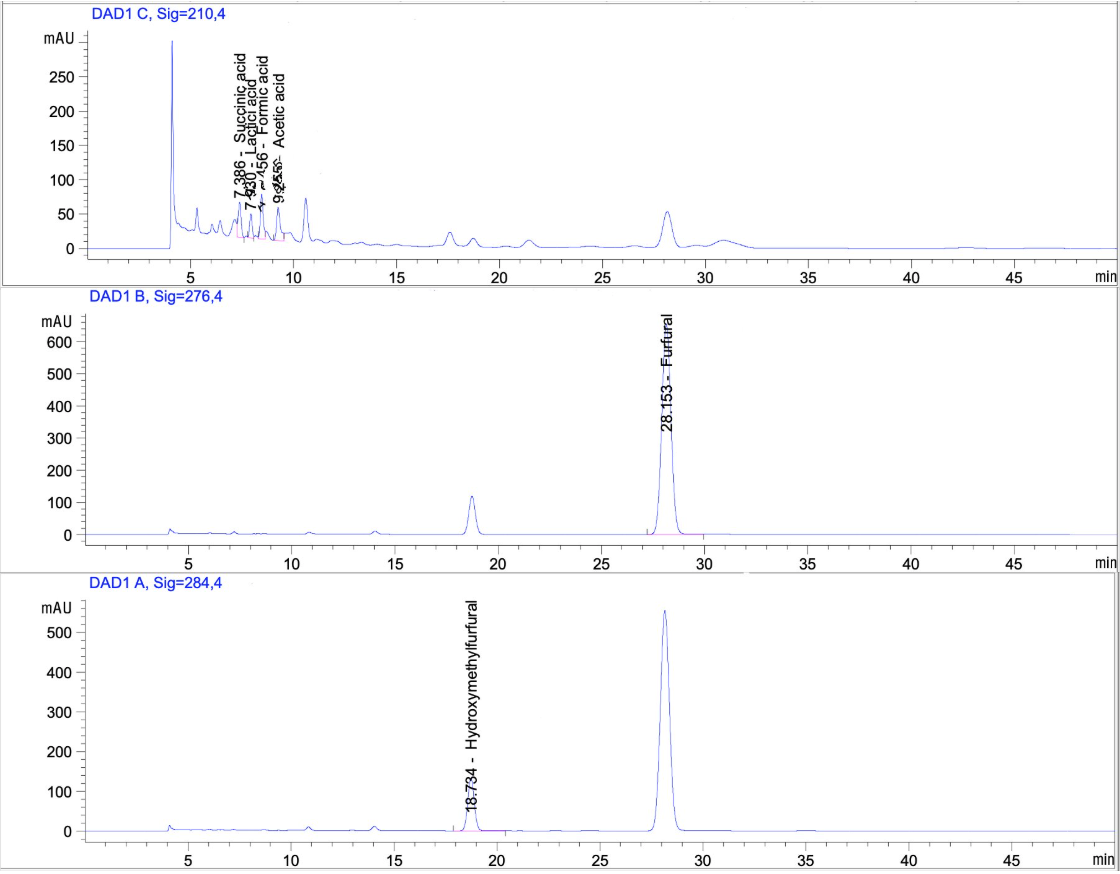


**Figure S11.** Chromatograms of the chromatographic conditions 1.0 ml/min at 60°C of the study sample wheat straw hydrolysate supplemented with 100 mg/l of succinic, lactic, formic and acetic acid, 3 mg/l HMF and 24 mg/l furfural

**References**

5 Świątek K, Gaag S, Klier A, Kruse A, Sauer J, Steinbach D. Acid hydrolysis of lignocellulosic biomass: Sugars and furfurals formation. Catalysts 2020; 10(4): 437. https://doi.org/10.3390/catal10040437

6 Li J, Xu Y, Zhang M, Wang D. Determination of furfural and 5-hydroxymethylfurfural in biomass hydrolysate by high-performance liquid chromatography. Energy Fuels 2017; 31(12): 13769-13774. <https://doi.org/10.1021/acs.energyfuels.7b02827>

7. Albouchi, A, Murkovic, M. LC method for the direct and simultaneous determination of four major furan derivatives in coffee grounds and brews. J. Sep. Sci. 2019;.42(9), 1695-1701. DOI: 10.1002/jssc.201900061

8 Xu C, Alam MA, Zhang J, Wang Z, Chen H, Xie C, Xu J. Identification of lignocellulosic derivatives inhibiting succinic acid fermentation and molecular mechanism investigation. Ind Crops Prod 2022; 187: 115466. https://doi.org/10.1016/j.indcrop.2022.115466

9 Casella P, Loffredo R, Rao MA, Balducchi R, Liuzzi F, De Bari I, Molino A. Inhibitors derived from wheat straw hydrolysate can affect the production of succinic acid by Actinobacillus succinogenes. Process Biochem 2024; 147: 228-239. https://doi.org/10.1016/j.procbio.2024.08.017

10 Hijazi A, Pisano I, Illek P, Leahy JJ. A rapid HPLC method for the simultaneous determination of organic acids and furans: food applications. Beverages 2022; 8(1): 6. https://doi.org/10.3390/beverages8010006

11 Saengsen C, Sookbampen O, Wu S, Seetasang S, Rongwong W, Chuaboon L. The potency of HPLC-DAD and LC-MS/MS combined with ion chromatography for detection/purification of levulinic acid and bio-compounds from acid hydrolysis of OPEFB. RSC Adv. 2022; 12(44): 28638-28646. DOI: 10.1039/D2RA03563D

12 Arti DK, Cheewasedtham W, Rujiralai T. Simultaneous quantification of volatile fatty acids and nonvolatile organic acids in Hevea brasiliensis latex. J. Sep. Sci. 2022; 45(18): 3491-3500. DOI: 10.1002/jssc.202200061
